# Trauma, Justice, and Equity: Using Critical Theories and Concepts to Address Systemic Harm Among Youth Punishment System-Involved Black Girls

**DOI:** 10.3390/bs15010031

**Published:** 2025-01-01

**Authors:** Camille R. Quinn

**Affiliations:** Center for Equitable Family and Community Well-Being, School of Social Work, University of Michigan, 1080 South University Avenue, Ann Arbor, MI 48109, USA; crquinn@umich.edu

**Keywords:** Black girls, youth punishment system, trauma, PTSD

## Abstract

This review critically evaluates the existing literature on youth punishment system (YPS)-involved Black girls and their intersections of with trauma and post-traumatic stress disorder (PTSD). It synthesizes findings from previous studies, identifying key research trends, gaps, and controversies, while also highlighting areas in need of further investigation. Black girls, particularly those involved in systems such as juvenile justice, child welfare, and education, often face disproportionate exposure to violence, abuse and neglect, trauma, and systemic racism. For Black girls with histories of trauma and PTSD, these intersecting challenges are compounded by the added vulnerabilities of race, gender, and YPS involvement. This article argues that addressing the complex needs of this population requires recognition that Black girls’ harm is criminalized and, therefore, inextricably linked to their YPS-involvement, so a comprehensive, culturally responsive approach that includes trauma-informed care, healing-centered engagement, and holistic support systems are needed. Equitable access to tailored mental health services, educational resources, and culturally relevant interventions is essential to mitigate the long-term effects of trauma, promote resilience, and foster healing. Additionally, advocacy efforts to dismantle systemic harm and address racial and gender disparities are critical for creating inclusive environments that empower and support Black girls in these systems. By centering their lived experiences, this review emphasizes the importance of fostering environments of healing, justice, and equity for this vulnerable population.

## 1. Introduction

In 2014, the New York Times published an opinion piece about mass incarceration being the greatest public health challenge of modern times based on a report released that same year (The Editorial Board, 2014; Vera Institute of Justice, 2014). Of note, in recent decades, women’s incarceration has grown at twice the pace of men’s incarceration and has disproportionately been located in local jails, warranting even greater concern (Vera Institute of Justice, 2014). In 2022, over 975,000 (though many more men are in prison than women, the rate of growth for female imprisonment has been twice as high as that of men since 1980 (Budd, 2024)) women were under the supervision of the U.S. criminal justice system, underscoring the extensive and disproportionate impact of incarceration on women (Budd, 2024). By 2023, the National Institute for Health Care Management (NIHCM) Foundation reported that 172,700 women were incarcerated, comprising approximately 10% of the total incarcerated population. This significant figure signals an urgent and deepening crisis of mass incarceration among women and girls, necessitating critical attention to the underlying systemic inequities driving these outcomes (Brinkley-Rubinstein, 2023). Briefly highlighting the arrest and incarceration rates and trends of women, especially Black women, provides essential context for understanding the experiences of Black youth and Black girls in particular within the youth punishment system—YPS. The structural and systemic factors that disproportionately affect Black women—such as racial and gender biases, poverty, and limited access to resources—are reflected in the challenges faced by Black girls (Ocen, 2015).

Black (or African American, we use the terms interchangeably in the U.S.; Quinn et al., 2023a; Slopen et al., 2010) women with histories of legal system involvement face a disproportionate burden of health challenges, including higher rates of sexually transmitted infections, HIV/AIDS, substance use disorders, and post-traumatic stress disorder—PTSD (Binswanger et al., 2012; Hatcher et al., 2009). Black women and girls face disparities rooted in a system that disproportionately criminalizes them for being Black, female, and poor, reinforcing punitive social control (Bach, 2013). Similarly, the health and mental health disparities faced by Black girls in the YPS are just as alarming and warrant attention (Fix & Mendelson, 2022; Rawal et al., 2004). In 2017, girls made up a disproportionately high 30% of youth arrests, despite only representing 15% of the youth detention population, signaling an urgent crisis within the youth punishment system (to reduce bias, stigma, and stereotypes associated with system-involved Black girls, the authors deliberately avoid the terms “juvenile offenders” and “juvenile justice system.” Instead, they use “youth” and “youth punishment system” throughout the text (Cole & Cohen, 2013; Kubek et al., 2020; Quinn et al., 2023a; Singh et al., 2020))—YPS (Dailey & Rosenbury, 2018; Quinn et al., 2023a). Notably, Black girls are disproportionately arrested, detained, and committed to residential facilities for status offenses and sent to adult prisons for violent offenses compared to their white counterparts (Patino, 2009). Structural and systemic factors contribute to higher rates of arrest and detention among Black girls, including adultification, racialized gender stereotypes, and the criminalization of their survival behaviors (Epstein et al., 2017; Marquardt, 2020; Tepper, 2014). The patterns of criminal justice involvement observed in Black women set a precedent for the obstacles encountered by Black girls, revealing the cyclical nature of racial and gender injustice. Understanding the plight of Black women illuminates how historical and ongoing inequities shape the YPS, creating compounded barriers for Black girls. This perspective underscores that early interactions with the criminal justice system are part of a broader trajectory of disenfranchisement that persists into adulthood, highlighting the urgent need to address these individual and structural and systemic issues across all life stages.

This review article presents theoretical and empirical evidence to emphasize the importance of Black girls in the YPS. Specifically, critical theories—Black feminist thought, critical race theory (CRT), and the intersectionality theoretical framework—are used to center Black girls. A gendered lens is essential for a comprehensive understanding of Black girls in the YPS, particularly through the complementary alignment of CRT and intersectionality. Both frameworks illuminate how race, gender, and other intersecting identities shape experiences of oppression and privilege. Rooted in legal studies, CRT and intersectionality facilitate a gendered analysis by examining how overlapping systems of oppression—racism, patriarchy, and capitalism—create unique challenges for women and girls of color (Bell, 1995; Crenshaw, 1991). For instance, a gendered CRT analysis may explore how racial stereotypes, such as the “angry Black woman” trope, shape workplace perceptions, while intersectionality emphasizes the necessity of addressing race within policies aimed at gender equality to ensure women of color are not excluded (Waller et al., 2022). Both frameworks have been employed by scholars to address racism, sexism, and classism, as exemplified by the work of Angela Davis and Audre Lorde. Davis highlights systemic and structural oppression, focusing on dismantling institutions, such as the prison industrial complex and advocating socialism to combat racial and gender inequities (Davis & Barsamian, 2000). She approaches intersections of race, class, and gender from a systemic and materialist perspective, prioritizing collective liberation over individual narratives (Davis, 2016). In contrast, Lorde emphasizes identity and lived experience, focusing on how intersecting identities, such as being Black, a woman, and queer, create unique forms of oppression. Her assertion that “the master’s tools will never dismantle the master’s house” critiques systems that fail to address the full complexity of identity and difference (Bowleg, 2021; Lorde, 1979/1984). While Davis’s work aligns with CRT through its systemic critique, and Lorde’s focus on identity aligns with intersectionality, their approaches collectively challenge the exclusion of race and gender in legal and feminist discourse. Together, they provide complementary perspectives for analyzing and addressing oppression. Moreover, all the critical theories are used to understand the criminalization of Black girls’ behavior, harm, (there is no universal definition of “harm” that describes Black girls’ or women’s experiences, particularly as a result of systemic issues like racial discrimination, gender bias, and criminalization. Some scholars use the term "gendered violence" as a comprehensive word that includes any form of harm inflicted without an individual’s consent, arising from power imbalances rooted in socially constructed gender roles, (Tonnesen, 2013)), so scholars, practitioners, and policymakers should strive to co-create environments of equity, justice, and healing when working with them (Williams-Butler et al., 2023). The Criminalization of Black Females is a new concept that is presented to show how their harm and need for help may serve as vehicles for punitive or racist treatment by system actors. This concept is further contextualized by regulatory intersectionality, the hyper-regulatory state, and the Circuits and Consequences of Dispossession to show how social services, law enforcement, and education systems adversely impact Black girls and women, especially those involved with the legal system. Overall, this article will play a significant role in advancing a scholarly understanding of these concepts by synthesizing and interpreting the existing research literature, providing context, and guiding future research efforts about legal system-involved Black girls with histories of trauma and PTSD.

## 2. Critical Theories

### 2.1. Black Feminist Thought

Black feminist thought (Collins, 2002) provides a critical lens for examining the intersections of race, gender, and class, particularly in understanding how systems of domination and oppression are structured and perpetuated. Collins (2002) outlines four interconnected domains of power within Black feminist thought, providing a robust framework to analyze the mechanisms of oppression and resistance. The first domain, Structural Power, refers to institutional arrangements such as education, legal systems, and labor markets that sustain systemic inequalities. The second, Disciplinary Power, encompasses the policies, practices, and bureaucratic processes that regulate individual behavior, reinforcing dominant ideologies and institutional hierarchies. The third, Hegemonic Power, centers on the cultural narratives and ideological frameworks that shape societal attitudes, perceptions, and norms, legitimizing existing systems of domination. Lastly, Interpersonal Power focuses on the micro-level interactions in everyday life, where power dynamics are reproduced or resisted within personal and communal relationships. These domains operate synergistically to uphold systems of oppression while simultaneously offering sites for contestation and transformation. Understanding their interplay is critical for interrogating the lived experiences of Black women, girls and other marginalized groups and for exploring how power is exercised and resisted in diverse contexts. This framework serves as a foundation for examining both the persistence of inequality and the potential for agency and collective resistance.

Collins (2002) also describes the “matrix of domination” which illustrates how interconnected social institutions—such as education, housing, employment, and government—create and maintain intersecting oppressions that disproportionately affect Black women and girls. This framework is especially relevant when considering the experiences of system-involved Black youth, who navigate multiple layers of marginalization, including those rooted in racism, sexism, and socioeconomic inequality. Domination operates through societal institutions that regulate and perpetuate patterns of oppression (Collins, 2002). Black girls involved in the YPS are particularly vulnerable to these intersecting oppressions, as they often experience systemic racism, sexism, and classism that shape their interactions with education, healthcare, and legal systems (Quinn, 2018; Quinn et al., 2023a; Waller et al., 2022). Researchers examining trauma in this population must recognize that their participants’ lived experiences are informed by these matrices of domination, influencing their perspectives on and interactions with institutional systems, i.e., the YPS.

Black feminist theory also underscores the importance of a bidirectional co-learning process between researchers and communities. Community-driven research must aim to create spaces where the voices of Black girls and young women are centered, and where both researchers and participants engage in mutual learning. This approach fosters a deeper understanding of the societal challenges that perpetuate trauma and oppression, enabling researchers to develop interventions that not only address individual trauma but also challenge the systemic forces that maintain domination and oppression. By using this framework, research on system-involved Black girls can more effectively contribute to health equity and the dismantling of oppressive structures.

### 2.2. Critical Race Theory—CRT

CRT, originally developed by Derrick Bell as part of legal scholarship following the Civil Rights Movement (Bell, 1995; Delgado & Stefancic, 2001; Roithmayr, 1999), has significantly shaped public discourse on race (Quinn & Grumbach, 2015). CRT critiques three key liberalist assumptions: (a) colorblindness is an effective solution to racism, (b) racism stems from individual actions rather than systemic structures, and (c) racism can be addressed without confronting intersecting forms of oppression such as sexism, homophobia, and economic exploitation (Valdes et al., 2002). Other scholars have challenged colorblindness, noting that it is ineffective at reducing racism and structural inequalities. For example, Ladson-Billings and Tate (1995) argue that educational disparities are deeply tied to racial injustices and require a comprehensive, systemic approach to resolve, emphasizing the importance of acknowledging and confronting race in educational policy and practice. Similarly, systemic inequities in the YPS may mirror education and the broader racial injustices in society.

Supporters of CRT emphasize social justice, centering marginalized voices and issues (Abrams & Moio, 2009; Solorzano & Yosso, 2001). By emphasizing the systemic nature of inequality and its intersections, CRT encourages a deeper exploration of how systemic racism contributes to trauma and adversity among Black girls in the YPS. CRT includes six tenets: (1) endemic racism, (2) race as a social construct, (3) differential racialization, (4) interest convergence/materialist determinism, (5) voices of color, and (6) anti-essentialism/intersectionality. Descriptions of each tenet can be found in other publications (Delgado & Stefancic, 2001; Quinn & Grumbach, 2015; Roithmayr, 1999). The intersectionality theoretical framework is the sixth CRT tenet. Specifically, intersectionality involves a combination of dynamics associated with different categories of identity, such as age, race, gender, sexual orientation, etc., that are most relevant in the case of young Black females (Crenshaw, 1991), which will be described in more detail in the intersectionality section of the review. It will be used to analyze and synthesize existing research findings to help researchers and practitioners stay current with developments in future research directions about Black girls and young women.

### 2.3. Intersectionality Theoretical Framework

The intersectionality theoretical framework focuses on how the experiences of marginalized people exist in multiple forms of interlocking aspects of social oppressions and the toll they exert on people of color. Specifically, it can be used to investigate how overlapping systems of oppression, such as racism and patriarchy, shape the lived experiences of individuals, particularly women of color (Crenshaw, 1991). This theoretical lens is especially relevant when examining harm and trauma among legal system-involved Black girls in the YPS, who often face compounding challenges related to race, socioeconomic status, and interactions with the legal system. Intersectionality is used as the organizing framework to understand the intersecting identities and the cumulative effects that impact the health of Black youth, especially Black girls (Gueta, 2020; Burgess-Proctor, 2006; Owen et al., 2017). The stress of trauma, like systemic oppression, accumulates over time and compounds disadvantages by reinforcing barriers such as racism and sexism—barriers that these systems often intensify. Black girls and young women experience multiple oppressions and harm that reinforce each other creating new categories of suffering (Aguilar, 2012; Taylor, 2019).

Intersectionality underscores the compounded sociological barriers of racism, capitalism, and sexism faced by African American women, often described as a “triple jeopardy” (Aguilar, 2012). The historic roots of intersectionality derive from the Combahee River Collective, a Black feminist organization founded in 1974, to highlight how overlapping oppressions create distinct forms of marginalization (Combahee River Collective, 1995; Crenshaw, 1991; Waller et al., 2022; Williams-Butler et al., 2024). Critical race theory, feminist legal theory, and Black feminist thought collectively inform Crenshaw’s (1991) conceptualization of intersectionality; to examine the ways race, gender, and class converge to shape the lived experiences of marginalized groups (Delgado & Stefancic, 2001; Hancock, 2013; Kolivoski, 2022; Waller et al., 2022; Williams-Butler et al., 2023). In the case of Black girls in the YPS, intersectionality provides a critical lens to understand the trauma they face prior to, during, and after they interact with the YPS, but within the context of broader systemic inequities. Crenshaw’s framework identifies three key dimensions: political, representational, and structural intersectionality. Political intersectionality indicates how individuals with multiple marginalized identities, particularly women (and girls) of color, can become entangled in the conflicting priorities of the different political groups they are part of. Also, political intersectionality highlights how the agendas of feminist and antiracist movements often marginalize the specific challenges Black women and girls face, resulting in insufficiently developed interventions for Black girls involved in the YPS (Waller et al., 2022). This further exacerbates their vulnerability, as culturally relevant mental health interventions remain scarce. Representational intersectionality critiques how Black women and girls are portrayed in popular culture, including the internet where racist and harmful stereotypes minimize their struggles and silence their voices (Waller et al., 2022). Further, representational intersectionality emphasizes how violence against girls and women of color is perpetuated through cultural representations and media portrayals, while also shedding light on how these women are further marginalized and silenced when communities of color defend such representations as cultural traditions or integral aspects of their identity. This dynamic reinforces intersecting oppressions, making it more difficult for them to challenge harmful narratives and advocate for their own well-being. By focusing on these intersections, representational intersectionality reveals the complex ways in which cultural defense mechanisms contribute to the marginalization of girls and women of color, further entrenching structural and systemic inequalities (Harris, 2020). Structural intersectionality explores how institutions such as the law enforcement/legal, education, and healthcare institutions perpetuate systemic disadvantages for Black girls, hindering access to services and support. This is especially relevant for understanding health and mental health disparities, as Black girls’ experiences are often overlooked or distorted in systems designed for more privileged groups. Structural intersectionality uncovers how socio-structural factors—such as poverty, racial discrimination, and institutional practices—disproportionately harm these girls. Building on Crenshaw’s framework, Bach (2013) introduces regulatory intersectionality, which characterizes how the functioning of a particular set of administrative structures, i.e., social welfare institutions targeting poor communities intersect with regulatory systems to share their information, thereby intensifying adverse consequences for noncompliance observed in welfare settings. By expanding the intersectional lens, this approach reframes systemic inequities as institutional failings rather than individual deficiencies, emphasizing the responsibility of social systems to rectify structural harms rather than marginalizing the affected populations (Baldry & McCausland, 2009; Baldry et al., 2013; Bunn, 2019).

Utilizing the intersectionality theoretical framework in research with Black youth and girls in the YPS allows for a more comprehensive understanding of the overlapping oppressions that contribute to their harm and trauma. Intersectionality highlights the unique suffering experienced by Black girls and women within a historical context, emphasizing how the denigration of Black female sexuality during slavery, the criminalization of Black women for moral offenses in the post-Civil War era, and the discriminatory practices of early juvenile reform institutions have contributed to the systemic injustices they face today (DeGruy, 2005). This historical framework is essential for understanding the ongoing challenges that Black girls encounter within the YPS (Ocen, 2015). This legacy of marginalization and structural harm continues to manifest in modern contexts, contributing to the ongoing trauma faced by Black girls who often encounter similar patterns of discrimination and criminalization within contemporary systems. Understanding these historical roots is essential for addressing the present-day challenges and traumas that disproportionately affect this population. It also encourages the development of nuanced, culturally responsive interventions that address their unique needs, facilitating both individual healing and broader systemic change. This approach is vital for advancing equity and justice in behavioral health and social services for marginalized youth populations.

### 2.4. The Criminalization of Black Females

Being poor, Black, female, and living in an urban setting provides the exact circumstances for individuals to experience first-hand the criminalization of needing help or support because “poverty focused social welfare programs employ the methods and modalities of the criminal justice system” (Bach, 2013, p. 18). Seeking support can paradoxically place Black girls and women at heightened risk of punitive consequences, including involvement in the criminal justice system. The hyper-regulatory state, characterized by mechanisms that disproportionately target individuals based on race, class, gender, and geography, often undermines meaningful responses to their needs (Bach, 2013). These mechanisms—encompassing criminalization, invasions of privacy, and regulatory intersectionality—function as tools of social control that perpetuate systemic subordination of marginalized communities, particularly poor Black girls and women. This process extends through inter-system information exchanges, where data flows from social welfare agencies to child protective services, and in some cases, the criminal justice system. Such exchanges magnify adverse consequences for individuals and their families, with each system reinforcing racialized and gendered disparities. Consequently, these overlapping regulatory structures disproportionately entangle African American families in cycles of surveillance and punishment, further entrenching inequality (Bach, 2013).

The criminalization (“Criminalization is the act of turning an activity into a criminal offence by making it illegal” (Stith Gambles, 2020, p. 31)) of Black girls has profound detrimental effects on their health, positioning it as a critical public health concern. Specifically, they experience racism that should be considered a critical factor given their reports of stress and trauma. Among young African American women, racism may exacerbate the negative effects of traumatic experiences (Danzer et al., 2016; Xu et al., 2024). For instance, a study found that lifetime racial discrimination predicted sexual risk behaviors in African American women in their mid-thirties (Pahl et al., 2023; Xu et al., 2024). In another study of African American young women, 44% of the sample reported experiencing stress due to racism and discrimination (Xu et al., 2024). It could be argued that criminalization is undergirded by racism. Specifically, it refers to the systemic processes by which the behaviors, vulnerabilities, and survival strategies of Black girls are disproportionately treated as criminal offenses rather than as expressions of unmet needs, harm, trauma, or structural inequities. This phenomenon is driven by intersecting forces of racism, sexism, and classism that drives discrimination and has been layered onto Black girls to contribute to higher rates of criminalization (Ocen, 2015). Further, building on Wacquant’s (2009) concept of the hyper-regulatory state, Ocen (2015) highlights how the historical intersections of racial and gender subordination—rooted in systems such as slavery and Jim Crow—have shaped a distinct and precarious conception of childhood for Black girls. This marginalized status exposes them to heightened criminalization and systemic neglect, denying them the full protections typically afforded to childhood.

Their unique vulnerability underscores how structural inequities perpetuate the disproportionate surveillance and punishment of Black girls within social and legal systems. Addressing it necessitates a multifaceted approach that includes education, policy reform, and research aimed at preventing harm and mitigating risks to health and safety. These strategies should focus on systemic change to reduce the cascading effects of criminalization, foster resilience, and support equitable access to resources for disease and injury prevention.

## 3. Selected Literature Review

The parameters of this review were included to address critical gaps in understanding the intersection of Black girls’ system involvement, especially the YPS and its broader implications. The selection criteria prioritized research based on social domains: individual, family, peer, school, community/neighborhood (Herrenkohl et al., 2000); however, it also includes structural and systemic inequalities, a new (sixth) domain that highlights macro-level factors that are relevant to Black girls in the YPS.

### 3.1. Individual—Black Girls’ Involvement in the Youth Punishment System

Legal system-involved Black youth are disproportionally affected by trauma and PTSD (Abram et al., 2004, 2013; Logan-Greene et al., 2017) that is directly associated with their contact with the YPS. Further, ongoing contact with the YPS and legal actors can be a traumatic experience for Black youth, as interactions with police and courts, detainment and incarceration, and aftercare often perpetuate trauma due to systemic racial injustices inherent in the juvenile justice system (Williams-Butler et al., 2023), with 70% of girls having histories of sexual or physical abuse suggesting “trauma-linked youth offenses and recidivism” (Meichenbaum, 2006; Kerig, 2018). In particular, 45% of justice-involved youth meet diagnostic criteria for PTSD (Rosenberg et al., 2014) compared to 5% of youth with no criminal justice involvement (McLaughlin et al., 2013). Girls involved in the YPS often endure significant adversity, including experiences of sexual abuse, homelessness, family trauma, and physical assault (Logan-Greene et al., 2017). Almost 10% of incarcerated girls in youth facilities are detained for status offenses, such as truancy and running away from home. The higher rates of PTSD among youth in the punishment system are associated with child maltreatment and increased stress (Agnew, 1985), especially for girls (Kerig, 2018). PTSD has also been linked with dysregulated HPA axis functioning (regulates cortisol) and increased inflammation that can lead to long-term physical health problems, exacerbating health disparities (Schauer et al., 2011; Volpe et al., 2017). About 40% of Black females report experiencing coercive sexual contact by age 18, reflecting a high rate of sexual trauma among girls of color (Kerig, 2018; Quinn et al., 2020b; Women of Color Network, 2006; Yoder et al., 2019). Victimization has been established as a stronger predictor of violence than severe mental illness alone for adults (Walters, 2011). In addition, Black girls are the fastest growing group in the YPS (Crenshaw et al., 2015; Morris, 2016; Office of Juvenile Justice and Delinquency, 2019; Quinn, 2024; Sherman & Balck, 2015) even though youth arrest rates have dropped to their lowest in four decades (Puzzanchera, 2021). Scholars note that Black girls’ delinquency is linked to various factors, including externalizing behaviors (Kalu et al., 2020), involvement in multiple systems like child welfare and the YPS (Quinn et al., 2017; Yoon et al., 2019), and experiences of violence, abuse, and maltreatment—all associated with trauma and PTSD. Girls are incarcerated for status- and survival-related offenses, such as running away and truancy, as well as sex work and petty theft, often committed after escaping abusive home environments (Tepper, 2014). Further, girls are disproportionately penalized for early sexual behavior. They are more likely to have experienced sexual assault prior to detention and to face incarceration for minor infractions stemming from societal expectations of how a “good girl” should behave (Gamal, 2017). Society and justice systems impose stricter behavioral expectations on all girls compared to boys; however, Black girls face the added burden of adultification, where they are perceived as older and more culpable, resulting in increased interactions with the YPS and more severe penalties (Kajstura, 2019; Epstein et al., 2017).

### 3.2. Family—Caregivers

The role of caregivers is crucial to the health equity, well-being, and healing of Black girls. Prior studies posit that weak parent–child relationships are salient among girls in the justice system (Keenan et al., 1999). Noting an absence of parental social support is associated with poorer overall well-being in Black girls (Bradley & Corwyn, 2000; Quinn et al., 2020b; Piko & Hamvai, 2010). In a national sample of adolescents and their parents, Black girls were less likely to display externalizing behaviors if they also reported high rates of parental attachment (Kalu et al., 2020). Support is not always positive (Quinn et al., 2020b); caregivers’ reactions to trauma may be most important to youth symptom presentation (Lieberman et al., 2005). Some youth may be compelled by caregivers to conform to behaviors that might cause them emotional distress and strain their relationship(s). The more distraught and diminished the caregivers’ coping, the greater the chance for adverse outcomes in youth exposed to trauma and/or abuse (Cicero et al., 2011). Parents who do not recognize child trauma symptoms often do so because of their own histories of trauma and current adulthood PTSD symptomology (Alisic et al., 2014). Further, children raised by caregivers with PTSD and traumatic histories (e.g., slavery and the Holocaust) may be genetically more susceptible to stress (DeGruy, 2005; Quinn, 2018; Shulevitz, 2014). Girls who experience problematic caregiver support could have greater PTSD symptoms (Quinn et al., 2020b). Black girls often rely on their caregivers for support, reducing their stress, PTSD symptoms, and delinquency, as well as improving their relationships (Huebner & Betts, 2002; Patterson, 2002; Werner & Silbereisen, 2003). These findings underscore the complex and multifaceted role of caregivers, highlighting the need for trauma-informed and culturally responsive approaches to support both Black girls in the YPS and their caregivers.

### 3.3. Peers

Peers play an important role in the lives of Black girls in the YPS (Crosby, 2016; Hubbard & Pratt, 2002; Quinn, 2014; Quinn et al., 2020b; Snyder et al., 2005). A study using Pathways to Desistance data (*N* = 163 girls, ages 14–18; 38% Black, 28% White, 31% Hispanic, and 3% Other) examined factors contributing to their criminal involvement, including perceived success, peer delinquency, parent–child closeness, and offense severity. Results showed that girls involved with delinquent peers were more likely to commit more severe offenses (Harlow, 2020). Associating with delinquent peers is a well-established contributor to adolescent delinquency, including gang involvement (Dishion et al., 2010; Haynie, 2001). Peer influence is a significant factor in shaping delinquent behavior, as gang membership often provides a context in which individuals engage in illegal activities and reinforce each other’s criminal behavior (Thornberry & Krohn, 2003). Further, youth gang members experience higher rates of PTSD symptoms than other youth in the justice system, and PTSD serves as both a consequence and a risk factor for continued gang involvement. Trauma exposure and post-traumatic stress symptoms are seen as contributing to gang participation and as a result of it, creating a cyclical relationship between trauma and gang involvement (Dierkhising et al., 2021; Harris et al., 2013; Kerig et al., 2016; Nydegger et al., 2019; Wojciechowski, 2021; Kerig & Mendez, 2022; Osman & Wood, 2018). The complex relationship between trauma, peer influence, and delinquency underscores the need for trauma-informed interventions that address underlying factors and prioritize healing for Black girls in the YPS.

### 3.4. School

Schools have been identified as precarious spaces for Black girls, particularly those engaged with YPS. Research highlights risk factors strongly associated with YPS involvement, including school suspension, expulsion, academic failure, parental incarceration, family conflict, histories of abuse, exposure to neighborhood violence, mental health diagnoses, and teen pregnancy (Gavazzi et al., 2009; Quinn et al., 2020b; Morris, 2016; Siegel & Williams, 2003; Thornberry, 2003; Trickett & Gordis, 2004; Zahn et al., 2008, 2010). Within the context of criminalization, school professionals often approach and respond to Black girls in ways that fail to recognize their age, mischaracterizing them as older and more mature than they are. This adultification bias contributes to significant trauma for Black girls and underscores their need for culturally competent and relatable interactions with supportive school professionals. The pervasive societal perception of Black girls and women as “combative” and “angry” (Morris, 2016) reinforces these harmful dynamics, fostering environments that exacerbate school pushout.

School pushout, primarily enacted through punitive disciplinary policies, reflects a dominant racist ideology that shapes how society interprets and responds to the behaviors of girls of color (Morris, 2016; Morris, 2018). Stereotypes portraying Black girls as “loud” or “angry” have become normalized, and even minor deviations from dominant expectations of femininity often prompt harmful responses from school professionals, including public embarrassment, excessive disciplinary measures, or physical aggression (National Women’s Law Center, 2016). These patterns highlight the urgent need for educational systems to adopt approaches grounded in cultural understanding, equity, and trauma-informed practices to dismantle the structural barriers disproportionately affecting Black girls. Consequently, it is of no surprise that Black girls account for more than one-third of all school-based arrests and law enforcement referrals, underscoring their disproportionate criminalization in educational settings (United States Office of Education, 2016).

Resistance to oppressive practices and policies is often a strategy employed by Black girls to protect and preserve themselves within academic settings. Behaviors such as assertiveness and defiance are frequently misinterpreted as non-compliance, non-conformity to gender norms, or intimidation, which often results in punitive responses, including suspension and expulsion (Morris, 2016). This systemic bias perpetuates the marginalization of Black girls within educational institutions. The existing literature calls for further exploration of the intersectional relationships between gender, culture, racism, victimization, and socialization to better understand their role in justice involvement among Black girls. Such analyses reveal the harms of treating girls as a homogeneous group, highlighting the critical need to investigate racial and cultural distinctions in their educational experiences (Ravoira et al., 2012). These perspectives advocate for nuanced and equitable approaches to addressing the unique challenges Black girls face in academic and disciplinary systems.

### 3.5. Community/Neighborhood

The neighborhood or community context significantly shapes adolescents’ behavioral outcomes and social development, with notable implications for Black girls involved in the YPS (Abrams & Freisthler, 2010). The quality of these neighborhoods, whether characterized by economic disadvantage or strong social cohesion—plays a critical role in influencing their experiences and trajectories (Kiser et al., 2010; Quinn et al., 2020a). For instance, research indicates that neighborhood cohesion can mitigate the effects of cultural race-related stress among Black families, primarily caregivers, by fostering a sense of procedural justice and reducing antagonistic perceptions of law enforcement. Such cohesion has been linked to improved mental health outcomes, underscoring the protective role of connected community networks (Quinn et al., 2020a; Quinn et al., 2023c). Conversely, neighborhood stress, often arising from systemic disinvestment, has been consistently associated with higher rates of delinquency and health risk behaviors among adolescents.

Black girls returning home from detention or other correctional settings frequently face substantial challenges in reintegrating into low-income neighborhoods (Abrams & Freisthler, 2010). These environments often lack essential resources, such as employment opportunities and rehabilitative support, which are exacerbated by structural barriers like the stigma of criminal records (Abrams & Freisthler, 2010; Loprest et al., 2019; Sampson et al., 2002). This disconnection from societal participation can perpetuate cycles of unemployment and reoffending (Loprest et al., 2019). Furthermore, these adverse neighborhood conditions are not isolated phenomena but symptoms of broader systemic processes, including structural racism and societal neglect (Dishion et al., 2010). The persistent under-resourcing of these communities reflects a larger pattern of disinvestment in marginalized populations, perpetuating inequities that disproportionately affect Black girls (Abrams & Freisthler, 2010; Dragomir & Tadros, 2020; Loprest et al., 2019; Sampson et al., 2002). Addressing these challenges requires systemic reforms that prioritize equitable resource distribution and foster supportive neighborhood environments.

### 3.6. Structural and Systemic Inequalities

Structural factors, such as poverty, violence, racism, and a general mistrust of mental health professionals create barriers to treatment access and are prevalent with Black girls in the YPS (Betancourt et al., 2016; Hockenberry & Puzzanchera, 2017; Lindsey et al., 2013; Sickmund & Puzzanchera, 2014). Poverty, along with dilapidated neighborhoods and low-performing schools, undermines community resilience to trauma and increases the likelihood of experiencing a history of trauma (Xu et al., 2024), especially for this population. A paradigm identified by Fine and Ruglis (2009) demonstrates how systemic policies confine Black girls by privileging certain racial groups, such as White and Asian populations. Consequently, the outcome for Black girls and young women, branding them as inherently less worthy. This framework, described as the “Circuits and Consequences of Dispossession,” delineates the processes by which marginalized groups—particularly poor, Black, and Latinx individuals—are systematically rendered disposable, portrayed as dangerous, and subjected to containment to safeguard their White counterparts. These systemic practices highlight racialized and gendered mechanisms of exclusion and control.

Structures that foster violence through social domains like interactions with malfunctioning or racist systems may cause Black girls harm and adversely affect their overall well-being (Kalu et al., 2020). Scholars note Black girls’ coping with harm, past trauma, and rejecting future victimization includes adopting an image of self-defense described as survival coping (Kerig, 2018; Goodkind et al., 2009; Morris, 2016). Self-defense is defined as self-protection and has been shown to help Black girls avoid danger (Jones, 2009). Also, personal agency reflects self-efficacy, how girls use their power, and their ability to engage in intentional decision-making based on accessible information (Boyd et al., 2020). Black girls have marginalized and intertwined identities that exist at the low end of the social hierarchy resulting in discrimination/racism and microaggressions (Potter, 2015; Waller et al., 2022; Quinn et al., 2023a; Quinn, 2024). They are often penalized and further harmed by teachers and other adults when they demonstrate self-protective behaviors, resulting in the coping strategies working in some contexts but not others, i.e., schools (Morris, 2016; Kalu et al., 2020; Mathies et al., 2020), so it is imperative to identify and understand these unique strategies.

Of note, African American women with histories of violence exposure, victimization, and intimate partner violence (IPV) often avoid formal service providers due to racism and routine mistreatment, including denial or delays in crisis interventions, highlighting systemic barriers to equitable care (Waller, 2021; Waller et al., 2023). Consequently, they are more likely to demonstrate trauma-related resistance that is often misinterpreted by providers who may exhibit bias and racism in their treatment of them (Quinn & Grumbach, 2015; Waller, 2021). Mistreatment and racism from service providers discourage African American IPV survivors from seeking help in the future, which increases their vulnerability and heightens the risk of femicide (Bent-Goodley, 2013; Waller et al., 2022; Waller et al., 2023). As noted here, the hyper-regulatory state broadens regulatory intersectionality, by describing mechanisms rooted in race (racism), class, gender, and geography that exert social control over poor African American women, girls, their families, and communities (Bach, 2013; Wacquant, 2009) and reinforcing structural inequalities.

Researchers and practitioners must consider the structural and systemic inequalities that Black girls face in the YPS because these factors are critical to understanding their disproportionate involvement and the specific forms of harm they experience. The overrepresentation of Black girls in the YPS cannot be fully explained by their individual actions but is deeply rooted in broader societal structures, including racial, gender, and class-based disparities (Morris, 2016; Gavazzi et al., 2009). Further, Black girls are likely to experience harsher treatment in the YPS, so they are more likely to remain in the system longer and experience more serious charges due to system actors’ gender stereotypes and biases, shaping how these professionals perceive and interpret girls’ actions (Anderson et al., 2023; Bridges & Steen, 1998; Graham & Lowery, 2004; Peck, 2018). School discipline policies, for example, frequently reflect racial and gendered stereotypes, such as the perception of Black girls as “loud” or “angry,” which leads to increased rates of suspension, expulsion, and referral to law enforcement (Epstein et al., 2017; Morris, 2016; National Women’s Law Center, 2016). These discriminatory practices push Black girls out of schools and into the YPS, where they are treated as adults and criminalized for behaviors that are often typical adolescent expressions of resistance or survival (Morris, 2016).

The systemic nature of these inequalities also involves broader social determinants, such as poverty, exposure to violence, family instability, and racial trauma, which compound the risk factors for Black girls in school settings (Gavazzi et al., 2009). Similar to Bach (2013) and Wacquant’s (2009) accounts of the perniciousness of social, education, and criminal/punishment systems, Black girls (and women) are more likely to be harmed than helped by these entities whether they are mandated to them or they seek out help on their own. These intersecting vulnerabilities are often ignored or misinterpreted within these systems, further perpetuating cycles of criminalization and marginalization. To effectively address the needs of Black girls in school, it is essential for researchers and practitioners to examine how these structural and systemic inequalities contribute to their involvement. Understanding and addressing the structural roots of their criminalization will enable more equitable interventions and policies that support Black girls’ well-being, rather than simply reinforcing punitive responses (Morris, 2016; Gavazzi et al., 2009). This approach not only improves outcomes for Black girls but also works toward dismantling the broader systems of racial and gender-based oppression that shape their experiences.

### 3.7. Disparities in Access to Services, Treatment, and Research

Black girls with mental health difficulties are less likely than other racial and ethnic minority groups to receive treatment (Lindsey et al., 2013). The number and scope of services for females is much lower than what is available for males (Van Voorhis et al., 2009; Van Voorhis, 2012). In essence, Black girls are less likely than White girls to receive treatment, even when they are in residential care (Mojtabai & Olfson, 2020). Even when they receive mental health services, their outcomes are mixed (Espinosa et al., 2013; Espinosa et al., 2020; Williams-Butler et al., 2023). Scholars have noted that girls with trauma experiences in the YPS were more likely to be funneled into the YPS compared to boys and those without a mental health need, including being rearrested (Espinosa et al., 2013; Williams-Butler et al., 2023). Black girls are underserved and understudied despite the need for innovative research that could promote their health equity (Quinn et al., 2020b).

## 4. Discussion

### 4.1. Recommendations for Black Girls’ Treatment

There is a notable lack of interventions tailored to the distinct cultural and developmental contexts of Black adolescent girls in the U.S. (Williams-Butler et al., 2023), including those in the YPS. In a scoping review of interventions for Black girls, four interventions were identified that were both culturally and gender-responsive, three interventions were primarily culturally responsive, and one intervention was primarily gender-responsive with all articles focusing on Black girls’ well-being. Several scholars are developing sexual health interventions to promote Black girls’ sexual and mental health equity (e.g., substance misuse), including girls in the YPS. Black girls face disproportionate rates of HIV and sexually transmitted infections (Boyd et al., 2020; Crooks & Muehrer, 2019; Crooks et al., 2023; DiClemente et al., 2014; Quinn et al., 2023a), are overrepresented in the YPS (González, 2018; Quinn, 2014; Logan-Greene et al., 2017; Kim et al., 2019a; Quinn et al., 2023a, 2023b; Kolivoski et al., 2023; Sherman & Balck, 2015), and experience substance misuse (Opara et al., 2022, 2023, 2024; Quinn et al., 2023b). One sexual reproductive health intervention with Black girls has produced numerous results that inform current and future interventions. Many of the studies included in this review are based on secondary data from the Imara parent study, a randomized controlled trial designed to evaluate the efficacy of a sexual risk reduction intervention. This study was funded by National Center for HIV/AIDS, Viral Hepatitis, STD, and TB Prevention (NCHHSTP) (5UR6PS000679–04) to test an intervention specifically aimed at reducing the incidence of sexually transmitted infections (STIs), promoting HIV-preventive behaviors, and improving psychosocial outcomes among Black girls in a juvenile detention center (Davis et al., 2016; DiClemente et al., 2014; Quinn, 2018, 2024; Quinn et al., 2020b, 2023a, 2023b). The complete efficacy study has been detailed elsewhere (DiClemente et al., 2014). Many of the study results note the strengths and protective factors of Black girls, including having high reports of self-esteem and future orientation despite their reports of PTSD (Quinn et al., 2020b), ability to discern unsafe intimate relationships with adverse partners (Quinn et al., 2023a), and that older Black girls abstain from substance misuse while engaging in sexual activity (Quinn et al., 2023b). All these studies highlight the urgent need for additional research to produce innovative strategies to improve intervention and prevention efforts.

### 4.2. Recommendations for Black Girls’ Research

Future research is critical to develop effective interventions that center on the distinct and intersectional challenges and strengths that Black adolescent girls experience within the YPS and society. More research studies are needed to assess the utility, effectiveness, and efficacy of tailored and culturally appropriate treatment for Black girls in the YPS. This is needed since they tend to be more vulnerable to adverse health and mental health outcomes and recidivism when compared to boys (Baglivio & Epps, 2016; Conrad et al., 2014; Craig et al., 2020). Research focused on Black girls who are court-involved but are living in the community is warranted given that they represent most girls who are also less likely to receive needed mental health services since they are not detained or incarcerated (Hockenberry & Puzzanchera, 2017; Lindsey et al., 2013; Sickmund & Puzzanchera, 2014; Tam et al., 2019). To optimize their health equity, scholars and researchers must identify prevention and intervention strategies with the potential for favorable outcomes. Recent efforts by the National Juvenile Justice and Delinquency Prevention Coalition’s (NJJDPC) suggest a trauma-informed approach and collaborative treatment efforts across child and family service organizations for youth with trauma histories (Olafson et al., 2016; Yoder et al., 2019). Many girls reside in high-need, under-resourced communities that are particularly susceptible to PTSD symptoms noting a push for criminogenic factors predicting criminal behavior, such as parent maltreatment, trauma, and addiction-based treatment of this population (Brown et al., 2020; Epperson et al., 2014; Liu et al., 2020; Quinn et al., 2020b; Walters, 2011). The impact of the proposed research is high with great public health significance via its potential to reduce and prevent PTSD and delinquency. Previous findings have been mixed for Black girls with trauma who receive public mental health treatment, as some are funneled into the YPS or their lengths of stay in correctional facilities increase (Espinosa et al., 2013; Espinosa et al., 2020; Williams-Butler et al., 2023). Consequently, matching services with their criminogenic needs—education and antisocial behavior to reduce future criminal behaviors—is warranted (Andrews et al., 1990; Andrews & Bonta, 2010).

Cognitive behavior therapies (CBTs) using exposure to directly confront trauma stories have been at the forefront of evidence-based treatments for PTSD, though they may not be well tolerated by some victims of chronic trauma (Nemeroff et al., 2006; Volpe et al., 2017). A review of exposure-based gold standard CBTs for PTSD noted patient intolerance with trauma exposure (Orsillo & Batten, 2005). In a test of narrative exposure therapy with homeless African American young adults, one female participant dropped out of the study because they could not recall their trauma history (Quinn et al., 2017). Mindfulness-based Stress Reduction (MBSR) interventions are focused on functioning and well-being to increase awareness of their sensory, cognitive, and affective responses as they arise and does not require exposure to their trauma stories (Dutton et al., 2013; Nemeroff et al., 2006; Stephenson et al., 2017). Consistent with the prevention and intervention focus of the National Juvenile Justice and Delinquency Prevention Coalition, current and future studies that use healing to culturally adapt MBSR and other Cognitive Behavioral Interventions are needed to promote their wellness and personal agency.

The intersectionality theoretical framework focuses on how the experiences of marginalized people exist in multiple forms of interlocking aspects of social oppressions and the toll they exert on people of color. Intersectionality will highlight the intersecting identities and the cumulative effects that impact the health of Black girls (Burgess-Proctor, 2006; Owen et al., 2017). The stress of trauma creates cumulative disadvantage linked to barriers like racism and sexism. Black females experience multiple oppressions that reinforce each other creating new categories of suffering (Aguilar, 2012; Taylor, 2019). Marginalized individuals and communities need approaches that will actively create environments where they can heal, grow, and lead change collaboratively (Hackett, 2013). In Teaching to Transgress, Hooks (1994) provide essential components of a transformative pedagogy that aims to liberate and uplift, particularly for marginalized individuals and communities. The concepts of critical reflection and dialogical engagement collectively offer a framework for self-exploration, connection, and activism. Critical reflection helps individuals see how their personal experiences intersect with broader social issues. Specifically, this is a practice where individuals examine their own lives and experiences critically, with an understanding of how larger systems of race, gender, class, as well as the YPS impact their identities and opportunities. Hooks encourages students and teachers to reflect on how their personal experiences are influenced by societal structures, which helps to uncover hidden biases and forms of oppression. For Black girls in the YPS and researchers, critical reflection is a tool for identifying internalized oppression and recognizing the systemic forces that contribute to inequality. Through critical reflection, individuals develop a greater understanding of their social contexts, which empowers them to address and resist oppressive structures in their lives. Further, dialogical engagement allows individuals to share these reflections in a supportive community, fostering understanding and building collective resilience. Hooks notes that true learning occurs in an environment where open dialogue is valued. Inspired by Paulo Freire, hooks argues that education, i.e., learning, should be a collaborative and dialogical process where all participants, especially those from marginalized backgrounds, feel empowered to share their voices and experiences, which reflect both CRT and intersectionality. This means breaking down traditional hierarchies during the research activities and promoting reciprocal learning, where Black girls and researchers both teach and learn. For marginalized communities, dialogical engagement creates space to express personal narratives and challenge dominant perspectives, fostering mutual respect and validation. This inclusive dialogue can be transformative, helping individuals feel understood and supporting collective healing and solidarity.

Healing-centered engagement—HCE (Ginwright, 2016, 2018)—has emerged as a useful approach to adapt current and new interventions. HCE is based on healing justice, a strengths-based framework to re-center culture and nurture well-being and involves transforming institutions and relationships to promote healing (Ginwright, 2016, 2018). HCE includes four main tenets: (1) cultural grounding—restoring identities, (2) asset-driven techniques, (3) participant and researcher healing, and (4) political/civic engagement, which aligns with MBSR, hooks, and an intersectional lens (Ginwright, 2016, 2018; Hooks, 1994). Critical reflection and dialogical engagement reflect HCE tenets—cultural grounding to restore identities is predicated on their ability to understand their social context in a supportive environment, respectively (Ginwright, 2016, 2018; Hooks, 1994). Intersectionality can also inform the development of MBSR interventions with mindfulness principles. MBSR study results note that learning mindfulness practices promote healing from trauma and reduces the daily stress of the general population youth with trauma histories (Dutton et al., 2013). Since Black girls are at increased risk for a variety of adverse outcomes, improved self-regulation and stress responses may produce substantial health benefits for them (Quinn, 2024). Further, the legal system presents challenges for intervention researchers, such that nonrandomized designs are warranted and have shown utility in their examination of program efficacy in an urban police department and family court and a state correctional program (El-Bassel et al., 1997; Schilling, 1997, 2010; Soydan, 2015).

Future research should also investigate the specific ways in which Black girls and young women experience harm and dispossession, with an emphasis on understanding their responses to oppression and their interactions with institutional systems. These lines of inquiry could illuminate pathways for resistance and the broader implications of systemic involvement in Black girls’ lives.

## 5. Conclusions

This review examines research on YPS-involved Black youth, with a focus on Black girls and their traumatic experiences. It identifies key trends, gaps, and unresolved issues in the literature, while highlighting areas for further investigation, i.e., the criminalization of Black female harm. Black girls in systems such as youth punishment, child welfare, and education face disproportionate exposure to harm, violence, neglect, trauma and systemic racism. For Black girls with histories of trauma and PTSD, these challenges are intensified by intersecting factors of race, gender, and system involvement. This review emphasizes the need for comprehensive, culturally sensitive approaches, including trauma-informed care, healing-centered practices, and tailored interventions that will enhance the incredible strengths they already possess. Equitable access to health and mental health care, educational resources, and culturally responsive services is critical to mitigating trauma’s long-term impacts and fostering strengths- and assets-based outcomes. Advocacy to dismantle structural and systemic barriers and address racial and gender disparities is crucial to creating inclusive, supportive environments that promote justice, equity, and healing for Black girls.
